# From the pitch to personal growth: Investigating self-esteem as a mediator and parental support as a moderator in youth sports in China

**DOI:** 10.1016/j.heliyon.2024.e31047

**Published:** 2024-05-10

**Authors:** Kai Yi, Han Luo, Lihong Wei

**Affiliations:** a Physical Education College, Hunan University of Technology, 412007, Zhuzhou, Hunan, China; b Changjun Bilingual School, 410013, Changsha, Hunan, China; c School of Physical Education, Changsha University of Science and Technology, 410076, Changsha, China

**Keywords:** Youth sports participation, Character development, Life skills acquisition, Demographic factors, Parental support, Peer influence, China

## Abstract

This study focuses on the intricate connections among youth sports, personal development, and skill acquisition in contemporary China. Building upon established theoretical frameworks, the study aims to unravel the intricate interplay among various variables. Employing a robust methodology that accounts for mediation and moderation effects and with a sample of 808 individuals representing diverse demographics, the findings shed light on the significant influence of family structure, particularly the prevalence of extended family arrangements, on an individual's character development. Furthermore, the study underscores the pivotal role of personal characteristics, particularly self-esteem, in shaping admirable traits. The research identifies several contributing factors to positive character development, including active participation in sports, parental support, accessibility to sports facilities, positive peer influence, and high self-esteem. Parents play a crucial role in mitigating the adverse effects of peer pressure by offering positive reinforcement and serving as role models. These findings hold important implications for youth development programs, emphasizing the vital role of parents in guiding character development, particularly in the context of peer pressure.

## 1 Introduction

Young individuals derive valuable lessons in character, collaboration, and life skills from their engagement in sports, extending beyond mere competitiveness and athleticism. Sports' widely recognized positive influence on children's development underscores its significance. Young people in structured sports get physical advantages and acquire essential lessons in teamwork, self-control, and persistence [1], which apply to their personal and professional endeavors. Youth sports have far-reaching beneficial impacts on society beyond the physical locations where they occur. Dorsch et al. [2] suggest that youth sports mold the identities of young individuals, imbue values, and impact the courses of their lives. They provide a secure environment for young individuals to acquire, hone, and assimilate essential life abilities. These skills surpass the technical components and incorporate emotional maturity, communication competence, leadership, foresight, and flexibility. Discipline, perseverance, respect, and empathy are essential character attributes for personal growth and societal well-being, all of which are nurtured by participation in sports [3].

The study's use of “Pitch” suggests that it alludes to several parts of the sports setting within the research framework. When determining how a youth's sports experience and growth are shaped, the four independent variables—sports facility, parental support, peer influence, and self-esteem—are vital. Because of its impact on accessibility and quality, a “Sports Facility” is where sporting events occur. Both “Parental Support” and “Peer Influence” illustrate how the social dynamics of the sporting environment impact the development of character traits, including drive, acceptance, and conduct. Finally, “Self-Esteem” includes individual psychological aspects like acceptance and competence that are impacted by sports engagement. To further understand how the sports environment, symbolized by “Pitch,” affects young outcomes, the research explores the mediating effects of these factors on character development.

Past studies have shown that mediation analyses must contemplate several liberated factors to fully understand the intricacy of interdependent factors. For instance, Slutzky & Simpkins [4] underlined the importance of studying parental involvement, peer interactions, and individual characteristics in their study on the role of sports participation in youth development. Understanding how sports engagement affects outcomes like self-esteem and social skills is vital. In order to understand how adolescent outcomes like academic achievement and well-being are impacted by environmental factors, personal attributes, and social influences, it is crucial to conduct mediation analyses incorporating multiple independent factors [5,6]. There is a need to learn more about the factors that influence adolescent growth and development if they look at parental support, peer interactions, and self-esteem all at once. Another rationale for including several factors in mediation studies comes from theoretical frameworks like Bandura's Social Cognitive Theory [7] and Bronfenbrenner's Ecological Systems Theory [8]. According to these models, human behavior and development are shaped by a complex web of interplaying personal traits, contextual elements, and social influences.

Youth sports activity is influenced by variables such as the availability of opportunities, peer and parental influence, and the accessibility of sufficient facilities. The changing connection between young athletes and their parents is further complicated by the athletes' confidence and the support they get [9]. The research intends to investigate the relationship between these variables, their impact on character development, collaboration, and life skills, and the influence of mediating and moderating factors in this context. Understanding these patterns among young individuals has societal and individual advantages. Engaging in sports enhances the overall health of young persons and better prepares them to face the difficulties of adulthood [10]. They are prone to demonstrating commendable character qualities, excelling in collaboration, and having a broad range of life skills that equip them to succeed in many contexts. These characteristics help create a more cohesive, empathetic, and unified community.

The sounds of youth athletics resonate uniquely across China's vast geography. In a nation that values its historical traditions as well as its contemporary advancements, sports play a complex, dynamic, and culturally significant role in the lives of young people. China is an exceptional place to study the significant relationship between youth sports and societal growth due to the intertwining of its historical grandeur and modern achievements [11]. Sports have far-reaching effects in today's multicultural and ever-changing society, shaping individuals, fostering collaboration, and imparting invaluable transferable skills to young people. The landscape of youth sports in the People's Republic of China is a stunning tapestry, rich in texture and character. China has a long and storied history with sports, evident from ancient martial arts to the global phenomenon of table tennis [12]. Basketball, soccer, and badminton, among others, have gained popularity due to the country's embrace of the Olympic spirit in this age of technology and global connectivity. Although sports enthusiasm spans demographics, it is most pronounced among young people. In an environment where tradition and modernity coexist, this study delves into the unique world of youth sports in China to shed light on how sports participation promotes character development, cultivates collaboration, and nurtures the acquisition of life skills. We draw on various ideas from psychology, sociology, and education as we explore the foundations of character development and life skill acquisition through sports in this narrative.

In China, children's sports hold significant importance. Sports in our country encompass more than just winning and losing; they reflect the nation's history and its aspirations for the future. In addition to enhancing their physical fitness, Chinese adolescents engaged in organized sports are also shaping their legacies, moulding their personalities, and acquiring the skills needed for success in the contemporary world [13]. People worldwide recognize that sports provide a well-rounded education that helps children develop essential life skills such as perseverance, discipline, collaboration, and empathy. As we delve into this investigation, we acknowledge that a myriad of factors naturally influences the dynamics of youth sports in China. Youth sports participation is heavily influenced by elements such as the frequency and intensity of participation, parental and peer involvement, and the availability of sports facilities [14]. Equally important in understanding this multifaceted relationship is young athletes' self-esteem and the support and encouragement they receive from their loved ones. Our research aims to shed light on how these variables interact, how they impact the development of character traits, collaboration, and life skills, and whether specific factors mediate or moderate this development. The complexity of this issue affects both the social and individual development of Chinese teenagers. Young people engaged in athletics are better prepared to face adversity, exhibit commendable qualities, collaborate effectively, and succeed in a dynamic and competitive society. In alignment with the country's broader goals, these qualities have the potential to contribute to a more peaceful, resilient, and empathetic society [15].

This investigation moves beyond theoretical study and delves into the realm of concrete data. Our objective is to collect information that represents the lives of young athletes, their families, and their communities. By doing so, we aim to influence policies, programs, and practices in China that promote the engagement of young people in sports in a manner that fosters their personal and social development. We explore not only the direct causal links between sports and positive traits such as self-control, perseverance, and collaboration but also the mediating and moderating variables that shape this connection. Crucial elements of our research include the mediating role of self-esteem and the influence of parental support and encouragement. In China's specific environment, these variables provide insight into how young athletes' experiences shape their social and developmental results. In conclusion, the Chinese youth sports landscape is a colourful and complex canvas, begging to be explored and understood. Our goal in delving into this subject is to shed light on the pivotal part of sports play for young Chinese people and to demonstrate the transformative potential of athletics in their lives.

Several research questions have developed during our discussion. Our primary goal is to investigate the role that parents play in shaping their children's character, teamwork, and life skills via their sports activities. In China, where youth sports are experiencing fast changes, it is essential to comprehend the cultural importance of these activities and how they relate to the development of admirable attributes, teamwork, and other transferable abilities in young people. Understanding how parental participation acts as a moderator in this connection is essential. Answering this question will help us comprehend how parental engagement and encouragement influence the positive developmental effects of children's sports. Second, we investigate how peer influences and the availability of sports facilities in China impact the development of life skills and character in young people. The availability of sports facilities may hinder sporting opportunities for young people in China's vast and diverse regions. Investigating the impact of peers on the experiences and outcomes of young athletes is crucial since peer influence is a dynamic factor likely to mediate this connection. Finally, we explore how self-esteem regulates the association between youth sports participation and improvements in character, collaboration, and life skills and how parental support and encouragement modify these effects. Nurturing young people's confidence through sports is vital. In China, it is essential to examine the role of self-esteem as a mediator between athletic engagement and positive outcomes, including personal growth, collaboration, and adaptability. Understanding how parental interest and involvement mediate these impacts is also essential since it influences the extent to which sports contribute to youth development in China.

The stated research questions are in line with the study's objectives, i.e..

I. To examine the relationship between the frequency and intensity of sports participation and the development of character, teamwork, and life skills in Chinese youth, taking into account the moderating influence of parental involvement.
II. To investigate the impact of the accessibility of sports facilities on the acquisition of life skills and character development in Chinese youth, with a focus on the mediating role of peer influence.
III. To assess the mediating effects of self-esteem in the relationship between sports participation and character development, teamwork, and life skills acquisition in Chinese youth, while exploring the moderating role of parental support and encouragement.

These research objectives guide the study's focus on understanding the interconnections between the variables and the roles played by moderating and mediating factors within the context of youth sports and development in China.

This study's importance rests on its attempt to tackle urgent issues related to the growth and engagement of young athletes in modern China. There is an urgent need to comprehend the complex ways in which Chinese youth sports impact their personal development, cooperation, and life skills in light of the country's fast cultural transitions and the changing nature of youth sports. Policymakers, educators, and practitioners working in t development programs might benefit greatly from the study's findings, which attempt to address gaps in the literature by exploring these dynamics. The importance of self-esteem in determining the results of young sports involvement is also becoming more acknowledged in the field of sports psychology, which is seeing rapid growth. Thus, this research aims to further our knowledge of the psychological processes driving adolescent growth in sports by investigating the mediating impacts of self-esteem. In the end, the study hopes to provide practical insights that help guide the creation of successful youth sports programs, which will promote the overall health and development of young people in China.

## 2 Literature review

The literature study is organized methodically, with three parts that each focus on a different major subtopic. Much research has investigated the complex link between participation in sports as a young person and the maturation of admirable character qualities; this section reviews some of these studies. This study establishes the groundwork for learning how youth sports engagement affects development in a variety of ways. The literature review switches its attention to the intricate relationship between youth growth and young people's access to sports facilities. This section explores the subtleties of how young people's entire development is affected by the availability and accessibility of sports facilities. It uses research and literature from a wide variety of fields to explain how easy access to sports facilities affects kids' physical, social, and mental development. Finally, the literature review wraps up with a deep dive into the mediating and moderating effects of self-esteem and parental support in the Chinese cultural context. This article sheds light on the complex interplay between a young person's sense of self-worth, parental encouragement, and other environmental factors in China.

### 2.1 Sports participation and youth development

Youth sports participation is a complex issue that has far-reaching implications for kids' maturation as people. Sports provide a crucial setting for the development of commendable traits, and the complex fabric of character growth highlights this. Youth who participate in organized sports report increased self-discipline, responsibility, and resiliency [16], all of which serve them well in later life. The world of sports is a fantastic laboratory for the development of these crucial personal qualities. Discipline is a fundamental trait for athletes who dedicate themselves to training and playing by the rules. The discipline and dependability they develop as athletes may be taken off the pitch and used in other areas of their lives [17]. In addition, the trials experienced in athletics are an excellent setting for developing fortitude in the face of adversity. Sports participation has been linked to the growth of life-enhancing traits, including perseverance, self-awareness, and a positive mental outlook [18]. Sports help shape people into better people because of the regulations and the direction of instructors. Young athletes benefit from the guidance of coaches who teach them not just the basics of the game but also the ideals of life. A coach's primary responsibility is to act as a role model for his or her players, teaching them not just how to win and lose gracefully but also the importance of integrity and fair play [19].

Youth sports are essential for building social skills and learning to work with others. Team sports are the finest way to see cooperation, communication, and leadership in action. When young people participate in sports, they are often exposed to complex cooperative situations in which they learn to work together to attain a shared objective [20]. In team sports, the value of cooperation is brought to the forefront. The success or failure of a group endeavor depends significantly on the team's capacity to work together, with each member being aware of how their contributions affect the overall. The importance of working together and getting along with others is emphasized in a variety of contexts [21]. Verbal and nonverbal communication skills are put to the test in the realm of physical competition. The ability to effectively communicate with one another while out on the pitch is crucial for relaying strategy, disseminating information, and fostering cohesive teamwork. Young athletes may benefit from developing their communication and listening abilities [22], which will help them not only share their thoughts but also comprehend those of their teammates and coaches.

Young individuals develop leadership, another critical aspect of collaboration, as they assume increasing levels of responsibility on sports teams. Young athletes learn to lead by setting an example for their peers, whether as team captains, mentors, or simply role models. Those who participate in athletics are more likely to become leaders in other areas of their lives, including their communities, schools, and places of employment [23]. Youth sports participation has several important outcomes, including character development, collaboration, and the acquisition of life skills. Sports provide a platform for young people to develop a wide range of abilities that help them navigate the intricate web of life. For instance, athletes sharpen their decision-making skills by constantly evaluating the pros and cons of specific actions in game situations. The ability to make quick decisions becomes a guiding compass for individuals when making choices about their future [24], whether it is related to education, work, or relationships. Time management is a vital part of engaging in sports and serves as a crucial life skill. Athletes learn time management skills by balancing various priorities, such as practice, games, and schoolwork. As they transition into adulthood, individuals can apply this skill to effectively manage their time between work, family, and hobbies [25]. The dynamic nature of athletics promotes adaptability, a key trait of resilient individuals. Athletes encounter a wide range of obstacles, adapt to changing playing conditions, and develop strategies to deal with the unexpected. Young people who acquire this ability are better equipped to handle life's uncertainties [26].

Athletes also develop problem-solving skills as they face tactical challenges and strategize to outsmart their opponents in sports. These problem-solving skills are crucial in today's society, whether in the workplace, personal relationships, or civic engagement [27], due to the increasing complexity of various aspects of life. Youth sports participation serves as a complex crucible where character development, collaboration, and life skills learning are nurtured. Young athletes cultivate a sense of accountability and tenacity through the ingrained discipline, responsibility, and resilience they acquire on the field. Team sports provide invaluable life skills in collaboration, communication, and leadership, and their impact extends well beyond the playing field. Young people need the tools to navigate the complexities of life, and they can gain them by learning how to make decisions, manage their time, be flexible, and solve problems [28]. In this rich context, sports serve not only as a platform for physical exercise but also as a significant catalyst for personal and social growth.Based on the comprehensive review of the aforementioned literature, the first research hypothesis is as follows.

H1 Youth sports involvement positively correlates with personal development among Chinese youth.

The acknowledged importance of athletic engagement in encouraging all-around growth provides the basis for this hypothesis.

### 2.2 Accessibility of sports facilities and youth development

The availability of sports facilities in China's vast and diverse regions is a critical factor that significantly influences the experiences and outcomes of children's sports participation [29]. The provision of well-maintained and easily accessible facilities substantially increases the likelihood of children participating in sports, laying the foundation for their personal and social growth through extracurricular activities. Given the vast size and population diversity of the country, Chinese researchers stress the importance of examining the impact of accessibility on young people's engagement in sports [30]. Young individuals are more likely to become actively involved in sports when they have convenient access to a wide range of sports facilities. In addition to enhancing skill development, this accessibility enhances the overall quality of sports-related experiences, which, in turn, encourages sustained participation [31]. There is a clear link between easy access to sporting facilities and the development of skills within the intricate framework of lifelong learning. Providing young people with convenient access to sports infrastructure significantly enhances their chances of acquiring a wide variety of life skills. This accessibility acts as a catalyst that not only improves life skills but also elevates the overall quality of sports experiences, promoting continued engagement [32].

Sports facilities provide an exciting environment for learning and practicing essential life skills. Young people engage in decision-making, a vital life skill, as they choose from among the numerous sports available to them. They have the freedom to select the sports they wish to participate in, determine how much time they devote to training and competition, and decide on the methods they will employ to reach their desired destinations [33]. Time management, another crucial life skill, is interwoven throughout the sporting experience. Young athletes learn to prioritize as they juggle multiple competing demands, such as training, schoolwork, and personal life. This skill, honed within the realm of athletics, serves them well as they transition into adulthood and learn to balance various responsibilities [34]. The ever-evolving nature of athletics fosters adaptability, a crucial trait in many facets of life. Adapting to changing playing conditions, making tactical adjustments, and dealing with the unexpected are just a few of the challenges young individuals must overcome. Sports instill in them the confidence, flexibility, and creativity to navigate the uncertainties in their professional and personal lives [35].

Athletes enhance their problem-solving skills by learning to adapt to evolving situations and devise innovative solutions to the challenges they encounter along the way. Young people who develop these skills through their involvement in sports are better prepared to address challenges in other aspects of their lives, including school, work, and relationships [36], compared to those who do not. The lives of young people are enriched when they have access to sports facilities, and this correlates with their increased likelihood of acquiring crucial life skills. Convenient access to clean, well-maintained facilities not only encourages more people to participate in sports but also enhances the overall quality of those who do. As a result, students become more engaged in their learning and acquire a myriad of valuable skills over time [37].

Especially in China's expansive and diverse regions, the availability of sports facilities plays a pivotal role in determining the experiences and outcomes of young sports involvement. These clean, accessible spaces are essential in attracting and retaining young people as active participants in sports. Moreover, they not only provide a conducive environment for skill development but also elevate the overall quality of sports experiences. Consequently, students develop skills including decision-making, time management, adaptability, and problem-solving, as well as a heightened sense of engagement [38]. Based on the comprehensive examination of the literature, the second research hypothesis is as follows.

H2 The accessibility of sports facilities is positively associated with the acquisition of life skills in Chinese children.

The significance of infrastructure in fostering youth development is discussed in this hypothesis.

### 2.3 Self-esteem as a mediator and parental support as a moderator: the role of social support nexus

Sports psychologists increasingly recognize the importance of self-esteem as a mediator in the connection between childhood sports and personal growth. The profound influence of sports on character development and life skill acquisition becomes apparent when we consider the positive impact of sports participation on young people's self-esteem. Sports serve as more than just a means of staying in shape; they provide an opportunity for self-discovery and personal growth. Young individuals often experience a boost in self-esteem and confidence as they become more involved in sports. Developing a healthy sense of self-worth is an ongoing process that thrives on the accumulation of minor triumphs along the way. This boost in confidence is not merely a byproduct of sports; it acts as a catalyst for further growth [39]. The cultivation of admirable qualities is intrinsically linked to a healthy sense of self-worth, serving as a foundation upon which other aspects of a person's personality can be built. Young individuals with a healthy sense of self-worth are more likely to practice self-control, resilience, and confidence. This self-assurance and adaptability in the face of change equip individuals for success in life [40].

Furthermore, self-esteem pervades all facets of growth, including but not limited to personality maturation. As one's sense of value and confidence rises, so does one's capacity to make wise decisions, organize one's time wisely, and adjust well to novel circumstances. These abilities are crucial for one's development and achievement in many areas of life [41]. When it comes to the connection between sports participation and young people's growth, the function of parental support and encouragement as a moderator is equally as important as the role of self-esteem as a mediator. A number of studies emphasize the importance of parents in shaping their children's sports careers [42,43]. Sports may have a favorable effect on children's self-esteem, character development, and general growth; however, this effect can be magnified or diminished depending on parental support. Parental involvement in their children's athletics, in the form of encouragement, attendance at games, and emotional support, might increase the favorable impact of self-esteem on character development. Their participation contributes to the establishment of conditions conducive to the growth of admirable characteristics. Synergistic effects on character development have been shown between suitable environments and the self-esteem gained via participation in sports [44]. Participation in athletics is an essential moderating element in children's development, particularly with regard to the acquisition of crucial character traits and practical abilities. This correlation may be considerably modified by parental encouragement and support; however, this is something to keep in mind [45]. Parental support increases the beneficial impacts of self-esteem on character development, producing a healthy atmosphere for teenagers to thrive in all areas via sports engagement. The third hypothesis of this study is as follows.

H3 The relationship between sports and personal growth is mediated by one's sense of self-worth.

New insights into the psychological mechanisms that motivate people to play sports are consistent with this hypothesis. The extensive body of research on the complex relationship between youth sports involvement and character development, teamwork, and life skill acquisition has shed invaluable light on these topics [46,47]. However, there are still evident gaps in the existing literature, as is common in any broad field of research. Firstly, most previous studies have primarily focused on Western cultural settings, giving less attention to the specific dynamics and effects at play in the Chinese context [48,49] than they should have. Given the stark differences between the educational and social landscapes in China and the West, it is crucial to explore how these factors manifest within the cultural context of China. The unique sociocultural elements that may influence the experiences and effects of youth sports involvement in China have generally been overlooked in existing research. Secondly, while previous research has touched on the roles of self-esteem and parental support as mediators and moderators, respectively, a more nuanced examination of these roles is warranted [50,51]. These elements are often presented as distinct influences on the relationship between childhood sports and maturation in the literature [52]. While self-esteem and parental support are two factors that may have a positive impact on a young athlete's performance, the interplay between these two factors has yet to be thoroughly explored in the existing research [53]. Additionally, previous studies have primarily focused on the causal links between sports and personal growth, teamwork, and skill development [54,55]. While these studies have provided significant foundational insights, they have yet to delve deeply into the underlying processes and pathways through which these effects are achieved. There remains a gap in understanding how various factors, such as the nature of the sport, the level of commitment required, and the influence of coaches, impact the development of character, teamwork, and other crucial soft skills in young athletes. This investigation, therefore, concludes that.

H4 The connection between parenting and character development is the influence of peers.

This hypothesis considers the complex role of social connections in determining the results for young athlete. This study was designed to supplement the current literature with a plethora of fresh information. As a first and foremost contribution to the current body of knowledge, this study examines the beneficial social and developmental consequences of juvenile sports participation with a particular emphasis on China and its unique setting. By doing so, we may learn more about the ways in which the Chinese social environment affects the development of young Chinese athletes' character, teamwork, and life skills. To maximize the beneficial effects of youth sports involvement in China, this information is crucial for designing culturally relevant programmes and interventions. Second, the study takes a multifaceted approach by looking at how self-esteem and parental support both act as mediators and moderators. It explores the intricate processes that determine the link between youth sports and general development by focusing on the interaction between these two aspects. Taking this stance allows us to hone our methods for encouraging virtue, teamwork, and the development of helpful life skills via physical engagement. Beyond the apparent correlations between sports activity and maturation results, this research goes further. It seeks to identify the underlying causes that are responsible for these results. The study's ultimate goal is to shed light on the many ways in which young people's exposure to sports influences their character, teamwork, and life skills.

## 3 Methods

### 3.1 Population, sample, and participants

The demographic breakdown of our sample is shown in Table 1. Schools, administrators, coaches, and instructors in the area of sports; parents and guardians; representatives in positions of authority; youth sports organizations; non-governmental organizations; specialists in the field of health and wellness; and media outlets all make up our study population. These groups can be found all around China, and all have their unique take on the role of sports in the development of China's children. Our study technique relied heavily on selecting a representative sample size. We carefully assessed the bare minimum number of replies we would need from each demographic to be confident in our results. We had a strong 77.69 % response rate from a total of 1040 questionnaires issued, demonstrating the participants' engagement and enthusiasm for the research. We targeted top universities and educational institutions in Beijing, Shanghai, Guangdong, and Sichuan as our primary target audience for educational institutions and administrators. To investigate the role of youth sports in educational institutions, we questioned 200 educators, coaches, and administrators. Forty different Chengdu, Shenzhen, and Xi'an-based sports coaches and educators were also contacted. These authorities provide light on the ways in which sports may help develop youth's character, teamwork, and well-being. Tianjin, Wuhan, and Chongqing parents and guardians were essential in our research. We questioned 600 people to learn more about parental support and participation in their children's sports. We also reached out to experts in the fields of health and wellness, government officials, policymakers, youth sports, and the media to get their take on the issue from a variety of angles.

**Table 1:** Target audience engagement, questionnaire distribution, and response rate.

Target Audience	Cities/Provinces	Organization names	Respondents	Relevance	Questionnaires distribution	Questionnaires returned	Response rate (%)
1. Educational Institutions and Administrators	Beijing, Shanghai, Guangdong, and Sichuan	1) Peking University 2 Renmin University of China 3) Beijing Normal University 4) Fudan University 5) Shanghai Jiao Tong University 6) Sun Yat-sen University 7) South China University of Technology 8) Shenzhen University 9) University of Electronic Science and Technology of China 10) Sichuan Normal University	Faculty, Staff and Administrators	Engage with educational administrators to understand the impact of youth sports participation on character development and life skills acquisition in their institutions.	200	157	78.5
2. Sports Coaches and Instructors	Chengdu, Shenzhen, and Xi'an	1) Chengdu Sports Bureau 2) Sichuan Sports University 3) Sports Training Centers in Shenzhen 4) Shenzhen Youth Sports Associations 5) Xi'an Sports Universities 6) Youth Sports Programs in Xi'an	Sports coaches and instructors	Gather their insights on how sports activities can contribute to character development, teamwork, and the acquisition of life skills among young athletes	40	28	70
3. Parents and Guardians	Tianjin, Wuhan, and Chongqing		Parents and Guardians	Investigate the level of support and involvement of parents in their children's sports activities.	600	485	80.8
4. Government and Policy Makers	Beijing, Guangzhou, and Hangzhou	1) Beijing Municipal Commission of Development and Reform 2) Beijing Youth Development Foundation 3) Guangzhou Sports Bureau 4) Guangzhou Municipal Education Bureau 5) Hangzhou Municipal Education Bureau 6) Hangzhou Youth Federation	Working members	Discuss how the research can influence policies related to sports infrastructure, youth development, and education.	40	29	72.5
5. Youth Sports Organizations	Shijiazhuang, Nanning, and Harbin	1. Shijiazhuang Youth Sports Development Center 2. Shijiazhuang Youth Sports Clubs 3. Nanning Youth Basketball League 4. Nanning Youth Swimming Club 5. Harbin Youth Sports Development Council 6. Harbin Youth Ice Hockey Association 7. Harbin Youth Taekwondo Club	Working members	Explore how they can enhance their programs to focus on character development, teamwork, and life skills alongside athletic training.	40	27	67.5
6. Community and Nonprofit Organizations	Qingdao, Changsha, and Zhengzhou	1. Qingdao Youth Sports Foundation 2. Qingdao Community Sports Clubs 3. Changsha Youth Sports Development Center 4. Changsha Youth Mentorship Program 5. Changsha Nonprofit Sports Organizations 6. Zhengzhou Youth Development Center 7. Zhengzhou Youth Empowerment Network	Working members	Discuss how the research can inform and improve sports-based programs aimed at character building and life skills development.	40	26	65
7. Health and Wellness Professionals	Fuzhou, Lanzhou, and Jinan	1. Fuzhou Wellness and Fitness Association 2. Fuzhou Sports Medicine Society 3. Fuzhou Health and Fitness Clubs 4 Lanzhou Wellness Centers 5 Jinan Sports and Fitness Clubs 6 Jinan Community Health and Wellness Organizations	Working members	Encourage the inclusion of sports and physical activities in wellness programs.	40	31	77.5
8. Media and Communication Outlets	Beijing, Shanghai, and Shenzhen	1. Beijing Sports Daily 2. Beijing Youth News 3. Beijing TV Sports Channel 4. Beijing Youth Sports Radio 5. Shanghai Sports News 6. Shenzhen Daily 7. Shenzhen Sports Broadcasting 8. Shenzhen Youth Sports Magazine	Working members	Encourage the media to raise awareness of the significance of youth sports participation and its impact on character development and life skills.	40	25	62.5
**Total**					**1040**	**808**	**77.69**

### 3.2 Sampling method

This research used a mix of convenience and purposive sampling methods. A purposeful sample strategy was used to reach specific demographics—including teachers, coaches, administrators, parents, and health and wellness specialists—because they were believed to possess knowledge and insight directly related to the study's aims. Using this method, we recruited people who could shed light on how child sports affect personal growth and health. Using convenience sampling, we contact more people by focusing on prestigious schools, locations with a strong reputation in youth sports, and elite colleges. Using this route, we efficiently and practically recruited participants while simultaneously collecting a wide range of viewpoints. The research grasped the intricate dynamics of Chinese adolescent sports because it used a mix of convenience and selective sampling methods.

### 3.3 Variables, measurement scales, and questionnaire items

The study's overarching goal is to learn more about how sports participation shapes young people's identities and equips them with valuable skills for later in life. To aid in this analysis, we have constructed a carefully built framework of components, each of which is crucial to understanding the nature of the connection under study.

#### 3.3.1 Dependent variable - character development (CHD)

Character development is a significant part of our analysis since it serves as a dependent variable. Children who participate in sports tend to develop in beneficial ways. To evaluate this idea, we use a 5-point Likert scale ranging from “strongly disagree” to “strongly agree.” The ‘Character Development Scale’ (CDS) was adapted to measure the maturation and character formation in young individuals due to their sports participation, and it served as the basis for item creation. Camiré and Trudel's [56] research on the social and moral character of sportsmen provided the foundation for this measurement.A sample question is presented as follows: *“I believe participating in sports has helped me become a better person.”* Duan et al. [57] employed a comparable scale and reported a Cronbach's alpha value of 0.76 for the internal consistency of the instruments.

#### 3.3.2 Independent variables

1. **Sports Participation (SPP):** This variable scrutinizes the regularity and intensity of youth involvement in sports activities. A 5-point Likert scale is deployed, adapted from the ‘Sports Engagement Scale (SES),' which measures the frequency and intensity of engagement in sports activities. The SES scale is an extension of the work by Sırgancı et al. [58]. An illustrative question is: *“I engage in sports activities multiple times per week.*” Qurban et al. [59] and Fan et al. [60] reported Cronbach's alpha values ranging from 0.74 to 0.96, suggesting a close relationship with the provided scale in mainland China.
2. **Parental Support (PAS):** This variable delves into the extent of parental support and active participation in their children's sports activities. A 5-point Likert scale is utilized, extended from the ‘Family Support Scale (FSS),' which gauges the degree of family support and active involvement in children's sports. The FSS scale is an extension of the scholarly work by Eime et al. [61]. An example question is: *“My parents encourage and support my participation in sports.”* Zhou et al. [62] and Wang et al. [63] have documented Cronbach's alpha coefficients falling within the range of 0.92–0.96. This range indicates a substantial alignment between the measured constructs and the provided scale within the context of mainland China.
3. **Sports Facilities (SPF):** This variable assesses the accessibility, affordability, and quality of sports facilities in the community. The ‘Sportscape Scale (SCS),' drawn from the scholarly work of Wakefield & Sloan [64], is employed for this purpose. An illustrative question reads: *“Are there safe and well-maintained sports facilities in your community?”* de Carvalho et al. [65] demonstrated a Cronbach's alpha value of 0.90 for the Sportscape scale in China.
4. **Peer Influence (PEI):** This variable explores the influence of peers on a young person's decision to participate in sports. The measurement scale used is the ‘Peer Motivational Climate in Youth Sport Questionnaire (PeerMCYSQ),' developed by Ntoumanis &Vazou [66]. It assesses the impact of one's social circle on sports engagement. A sample question is: *“My friends encourage me to participate in sports.”* The Cronbach's alpha value of the provided scale is approximately 0.90, as reported in the study by Çağlar & Kazak Çetinkalp [67].

#### 3.3.3 Mediating variable- self-esteem (SEE)

Self-esteem serves as a mediating variable, potentially influencing the relationship between the independent variables and character development. It measures participants' self-worth and self-perception, which their experiences can influence in sports. The ‘Behavioral Regulation in Sport Questionnaire (BRSQ)' is employed to gauge intrapersonal strength and well-being influenced by participation in sports. This scale is an extension of the scholarly work by Lonsdale et al. [68]. An example question is: *“My self-esteem is positively affected by my sports achievements.”* The scale's Cronbach's alpha value, as indicated in Liu et al.'s [69] study, is approximately 0.84.

#### 3.3.4 Moderator variable – social support nexus (SSN)

The study used ‘social support nexus’ as moderator of the study, which is formed through the combined effect of peer influence and parental support factors, incorporating characteristics of both the variables. It offers insights into the interplay between peer influence and parental support within the context of self-esteem development.

**Fig. 1:**
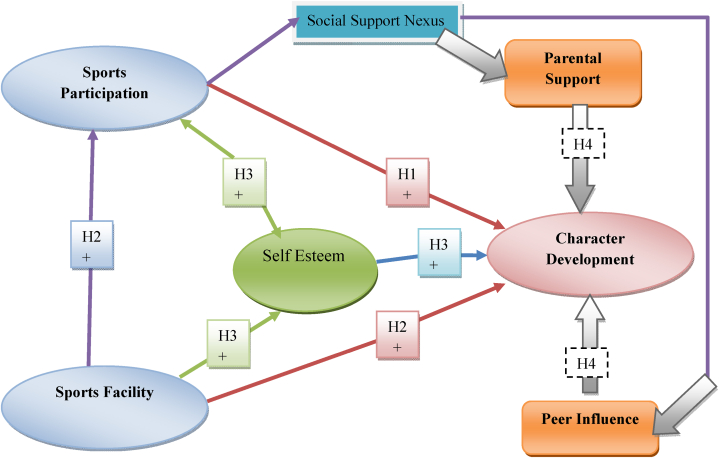
Theoretical framework.

Source: Author's extract.

These meticulously crafted variables, measurement scales, and questionnaire items collectively form the bedrock of our research methodology. They enable us to conduct a comprehensive investigation into the intricate relationships between youth sports participation, character development, and the associated factors, guiding our exploration with precision and clarity. Fig. 1 shows the theoretical linkages between the variables to support the hypotheses above.

The study asserts that self-esteem is a mediating variable in the relationship between sports involvement and personal development outcomes. Self-esteem is a separate factor that influences the connection between engaging in sports and other elements of human development, like character-building and acquiring life skills. The research suggests that self-esteem should be considered a mediating variable in the model since it represents the psychological processes via which sports engagement affects personal development outcomes. The research presents the following theoretical hypotheses based on it.

H5 Self-esteem is an intermediary between sports involvement and personal development results. Increased sports participation is linked to higher self-esteem, which subsequently results in enhanced personal growth, encompassing character development and acquisition of life skills.

This hypothesis is based on social cognitive theory [70], which suggests that involvement in activities like athletics might improve self-efficacy beliefs and, in turn, self-esteem. Furthermore, self-esteem is believed to have a role in other facets of human development, such as cultivating adaptive characteristics and abilities. The research argues that self-esteem plays a crucial role as a mediator in the connection between sports involvement and personal development results.

### 3.4 Statistical framework

#### 3.4.1 Data collection

The foundation of this research involved the meticulous collection of data to present a comprehensive view of how childhood sports impact personal growth and skill acquisition. Our study included participants from diverse demographics, encompassing higher education institutions, youth sports organizations, community and nonprofit groups, health and wellness experts, the media, and communication channels. We employed a variety of survey methods to engage these influential individuals, always taking care to safeguard their privacy and maintain the integrity of their data. Data were gathered diligently through a combination of online and in-person questionnaires, recognizing the need to accommodate our participants' interests and convenience.

#### 3.4.2 Data cleaning

Thorough data cleansing ensured that the gathered data was accurate. There were many moving parts to this, including careful management of gaps in the data. Careful consideration was given to dealing with missing data in surveys by using appropriate imputation techniques or, in the event of severely missing data, by excluding them from the analysis. In addition, we worked to find and deal with outliers as part of our data-cleansing efforts. The possible effect of these outliers, which frequently reflect data points drastically departing from the mean, was carefully examined. We used discretion when deciding whether to retain, transform, or eliminate outliers, always keeping in mind the broader context and the goals of our research. Another crucial part of our data-cleaning procedure was the validation of data inputs, which guaranteed that answers stayed within the specified margin of error and were consistent.

#### 3.4.3 Descriptive statistics

We initially examined the dynamics of our data through descriptive statistics, which is the traditional first step in data analysis. At this stage, we conducted a thorough investigation into the influence of peers on sports participation and self-esteem, as well as the frequency of youth engagement in sports and parental involvement in athletics. We calculated central tendencies such as means and medians to determine the average responses. In addition to this, dispersion measurements like standard deviations were used to provide an understanding of the data's range. Furthermore, we constructed frequency distributions to visualize how responses were distributed across various categories.

#### 3.4.4 Correlation analysis

We conducted a correlation analysis to delve deeper into the intricate dynamics that influence the variables in our study. Our objective was to gain insight into how the numerous factors impacted character development, making this study of utmost importance. We calculated correlation coefficients, with a particular emphasis on the Pearson correlation. These coefficients revealed the strength and direction of the relationships under investigation.

#### 3.4.5 Regression analysis

Regarded as a linchpin of our statistical analysis, multiple regression analysis was brought to the fore to explore the relationship between the independent variables, including Frequency of Sports Participation, Parental Involvement in Sports, Access to Sports Facilities, and Peer Influence on Sports Participation, and our dependent variable, Character Development. This form of analysis is uniquely poised to unveil the extent to which each independent variable contributes to the variance observed in Character Development, while duly controlling for other pertinent factors. Thus, we were able to investigate the pivotal question of how Frequency of Sports Participation, Parental Involvement in Sports, and other critical elements shape the development of positive character traits in youth.

#### 3.4.6 Mediation analysis

An essential phase of our inquiry involved mediation analysis, which is crucial in determining the role of self-esteem as a mediator in the relationships between independent factors and character development. In this investigation, our goal was to ascertain whether self-esteem serves as a mediating factor between the independent factors and the outcome of character development.

#### 3.4.7 Moderation analysis

The examination of moderation played a vital and theoretically intricate role in our research. It aimed to investigate how parental encouragement and support might mediate the relationship between peer pressure to participate in sports and low self-esteem. This analysis utilized interaction terms in regression analysis to carefully assess whether parental support and encouragement significantly moderate the influence of peers on self-esteem. This comprehensive examination enhanced our understanding of how parents can enhance their children's self-esteem within the context of sports involvement.

We exercised great care and precision in interpreting the findings from our statistical analysis. This stage involved a thorough analysis of the results, considering their magnitude, significance, and potential implications. These results carry both theoretical and practical significance beyond their statistical importance. We delved deeply into both theoretical and practical implications, where the former pertains to advancing scholarly discussions in the realm of youth sports and character development, and the latter concerns translating research findings into actionable insights for relevant stakeholders. We also ensured that our results were situated within the broader framework of our study's objectives and assumptions.

The study's methodology is designed to be as rigorous as practicable so that the results can be reproduced and used. Using the sampling procedure, a diversified sample of 808 people from different demographic backgrounds was recruited. This ensured that the sample adequately represented the target community of Chinese youth participating in sports. Transparency and future replicability were goals in providing detailed information about the sampling approach, which included inclusion and exclusion criteria, recruiting techniques, and sample characteristics. Participation in athletic activities, levels of personal development, and the impact of parents were some of the critical factors assessed using standardized assessments administered as part of the study's organized data-gathering method. For the sake of openness and replication, the study also meticulously records details on data-gathering tools, administration procedures, and any ethical concerns. To account for mediation and moderating effects, robust approaches were used in the statistical analyses done using multivariate regression by using SPSS and Amos software. The statistical methods further used, such as mediation and moderation analyses, are described in detail so that other researchers can reproduce and use the results.

## 4 Results

Table 2 presents a comprehensive summary of the demographic information for the participants in the Chinese sports and personality development study. The table displays the demographic breakdown of our sample, offering insights into the diversity and distribution of our respondents.

**Table 2:** Demographic survey.

Variables	Factors	Frequency (numbers)	Percentage (%)
Gender	Female	358	44.3
	Male	450	55.7
Age	>18–25 year	246	30.4
	26–33 year	158	19.6
	34–41 year	41	5.1
	42–49 year	176	21.8
	50 year and above	187	23.1
Education	Undergraduate	288	35.6
	Graduate	366	45.3
	Postgraduate	154	19.1
Geographical Status	Urban	522	64.6
	Rural	286	35.4
Family Structure	Nuclear family	304	37.6
	Extended family	398	49.3
	Single-parent household	106	13.1

The percentage of males in this category is somewhat higher than the percentage of females, at 55.7 %–44.3 %. Keeping things even-keeled is essential when thinking about possible disparities between the sexes in character development via athletics. There is a wide variety of ages represented, although the largest cohorts are young adults (18–25; 30.4 %) and middle-aged adults (26–33; 19.6 %). There was a wide range of ages represented among the participants, which may have affected the results on the impact of age on character development. There is a wide variety of educational attainment among the participants; 45.3 % have earned graduate degrees, 35.6 % have earned bachelor's degrees, and 19.1 % have earned master's or doctorate degrees. The possible impact of education on character traits requires data from a wide range of educational backgrounds. While the majority of participants are city-dwellers (64.6 %), a sizeable minority (35.4 %) hail from more rural places. This wide range of locations offers the possibility of illuminating differences between urban and rural environments with regard to character formation and access to recreational opportunities. There is a wide range of family structures represented among the participants: nuclear families (37.6 %), two-parent households (23.3 %), and one-parent households (13 %). Character formation and parental involvement may be affected by variations in family composition.

Descriptive statistics and Cronbach's Alpha values are summarized in Table 3 for the study's most important variables, shedding light on the study's fundamental tendencies, variability, and internal consistency.

**Table 3:** Descriptive statistics and Cronbach's alpha estimates.

Variables	N	Minimum	Maximum	Mean	Std. Deviation
Character Development (CHD)	808	1	5	3.668	0.879
Sports Participation (SPP)	808	1	5	3.647	0.902
Parental Support (PAS)	808	1	5	3.668	0.908
Sports Facility (SPF)	808	1	5	3.663	0.908
Peer Influence (PEI)	808	1	5	3.654	0.915
Self Esteem (SEE)	808	1	5	3.656	0.907
Social Support Nexus (SSN)	808	1	5	3.858	0.912

**Item-Total Statistics**					
**Variables**	**Scale Mean if Item Deleted**	**Scale Variance if Item Deleted**	**Corrected Item-Total Correlation**	**Squared Multiple Correlation**	**Cronbach's Alpha if Item Deleted**
CHD	18.2899	17.199	0.880	0.775	0.950
SPP	18.3111	17.034	0.877	0.776	0.950
PAS	18.2899	16.987	0.878	0.775	0.950
SPE	18.2953	17.059	0.866	0.751	0.951
PEI	18.3047	17.074	0.855	0.737	0.953
SEE	18.3025	17.087	0.863	0.746	0.952
SSN	18.2986	17.215	0.889	0.778	0.951

On a scale from 1 (Strongly disagree) to 5 (Strongly agree), participants scored an average of 3.668 with a standard deviation of 0.879. Both the mean and standard deviation for Parental Support (PAS) are 0.902, whereas the mean and standard deviation for Sports Participation (SPP) are 3.647 and 3.668, respectively. Similarly, the means and standard deviations for Sports Facility (SPF) and Peer Influence (PEI) fall within this range, at 3.663 and 3.654, respectively. These variables represent the ratings provided by participants on a 1 to 5 scale. Likewise, Self-Esteem (SEE) and Social Support Nexus (SSN) has a mean score of 3.656 and 3.858, with a standard deviation of 0.907 and 0.912, respectively, with response levels ranging from 1 to 5.Cronbach's Alpha estimates are presented for each variable in Table 3, confirming the validity of the instruments used. These estimates ensure that the scales effectively measure the intended constructs. The table demonstrates that if any item were removed from the Character Development (CHD) variable, the mean and variance would be approximately 18.2899 and 17.199, respectively. To assess how well each item correlates with the overall scale, we can examine the item's adjusted item-total correlation, which in this case is 0.880. It means that 77.5 % of the variation in this item is accounted for by the overall scale, as measured by the squared multiple correlation. Cronbach's Alpha, a measure of scale consistency, would decrease to 0.950 if any item were eliminated. The SPP and PAS variables follow a similar pattern, with almost identical data. Both of these variables exhibit excellent reliability (0.950) if any one item is removed and substantial corrected item-total correlations (0.877 and 0.878, respectively). The SPF variable has a lower squared multiple correlation (0.751) and adjusted item-total correlation (0.866). Nevertheless, even if one item were removed from this variable, Cronbach's Alpha would still be relatively high at 0.951, indicating excellent internal consistency. The PEI variable displays high adjusted item-total and squared multiple correlations of 0.855 and 0.737, respectively. After removing one item, Cronbach's Alpha remains relatively high at 0.953, demonstrating the scale's consistency. The SEE and SSN variables exhibits a similar corrected item-total correlation of 0.863 and 0.889 with a squared multiple correlation of 0.746 and 0.778, respectively. The scale's reliability remains strong, with a Cronbach's Alpha of 0.952 and 0.951, even when an item is eliminated.

An examination of the correlation matrix presented in Table 4 reveals the critical correlations between research variables.

**Table 4:** Correlation matrix.

Variables	CHD	SPP	PAS	SPF	PEI	SEE	SSN
CHD	1						
SPP	0.813***	1					
PAS	0.800***	0.826***	1				
SPF	0.796***	0.806***	0.800***	1			
PEI	0.807***	0.771***	0.780***	0.780***	1		
SEE	0.798**	0.788***	0.801***	0.781***	0.784***	1	
SSN	0.754**	0.721***	0.798**	0.745**	0.761***	0.745**	1

Note: *** and ** indicates 1 % and 5 % significance level.

With a correlation value of 0.813, it is evident that CHD and SPP are positively correlated. This discovery suggests that adolescents who regularly participate in sports exhibit signs of healthy character development. From a managerial perspective, this implies that initiatives promoting consistent youth sports engagement may contribute to enhanced character development, and educational institutions or sports organizations should consider designing such programs. Similarly, PAS demonstrates a significant positive association with both CHD (0.800) and SPP (0.826). This indicates that parental involvement and support for their children's sports activities have a positive impact on both their children's character and their likelihood of engaging in sports. Positive character development and increased sports participation may result from parental involvement in young sports, so policymakers should consider this when planning family-oriented programs. Although the association between SPF and the other variables is positive and statistically significant, it is somewhat weaker than those described above. The strong correlations between SPF and CHD (0.796) and SPP (0.806) suggest that increased community access to sports facilities may have a positive effect on character development and sports participation. Investing in the construction and accessibility of sports facilities may be considered as a means to enhance character development and increase sports participation among children. Both CHD (0.807) and SPP (0.771) are highly correlated with the PEI variable, indicating that peer encouragement to participate in sports positively affects both a young person's character and their level of commitment to the activity. By promoting a positive sports culture and peer networks, organizations and schools can harness the potential of peer influence to enhance students' character development and sports engagement. CHD (0.798), SPP (0.788), PAS (0.801), SPF (0.781), and PEI (0.784) all exhibit substantial positive relationships with SEE, underscoring the significance of factors such as parental encouragement, easy access to sports facilities, and positive peer influence in building one's self-esteem. Finally SSN exhibit a positive correlation with the CHD, SPP, PAS, SPF, PEI, and SEE. Coaches can positively impact young character development and sports participation by considering programs that enhance self-esteem through sports participation and provide support in various areas.

The Quartimax technique with Kaiser Normalization yields the Rotated Component Matrix, as shown in Table 5. This matrix illustrates the relationships between the survey questions and the underlying variables.

**Table 5:** Rotated component Matrix(Quartimax with Kaiser normalization) estimates.

Items	Component		
	1	2	3
CHD1	0.638	0.633	−0.015
CHD2	0.619	−0.010	0.007
CHD3	0.724	−0.151	−0.024
CHD4	0.677	0.038	−0.011
CHD5	0.689	−0.197	0.360
SPP1	0.667	−0.102	0.345
SPP2	0.673	0.180	0.249
SPP3	0.692	0.101	−0.094
SPP4	0.718	−0.104	−0.032
SPP5	0.671	0.024	−0.061
PAS1	0.687	0.029	0.404
PAS2	0.633	0.118	0.408
PAS3	0.691	0.173	0.151
PAS4	0.698	−0.096	0.066
PAS5	0.705	0.140	−0.149
SPE1	0.687	−0.050	0.007
SPE2	0.690	−0.072	−0.024
SPE3	0.681	0.038	−0.211
SPE4	0.686	−0.046	−0.066
SPE5	0.696	−0.082	0.088
PEI1	0.664	0.609	0.024
PEI2	0.690	0.003	−0.187
PEI3	0.687	0.017	−0.235
PEI4	0.673	0.062	−0.041
PEI5	0.697	−0.014	−0.109
SEE1	0.667	0.158	−0.002
SEE2	0.663	−0.076	−0.187
SEE3	0.675	−0.025	0.147
SEE4	0.703	−0.111	0.069
SEE5	0.686	0.051	−0.005
SSN1	0.668	0.045	0.004
SSN2	0.669	0.034	0.002
SSN3	0.684	−0.084	0.012
SSN4	0.681	0.024	0.018
SSN5	0.676	0.035	0.014

CHD-related items exhibit high loadings on Component 1. Several CHD scores, including (0.638), (0.619), (0.724), (0.677), and (0.689), demonstrate positive loadings on this component, indicating a strong relationship between these elements and the first component. There is a significant association between the assessment of youth character development through sports and Component 1. Component 2 may also load SPP items. Positive loadings on this component are observed for SPP1 (0.667), SPP2 (0.673), SPP3 (0.692), SPP4 (0.718), and SPP5 (0.671). This evidence suggests that Component 2 is linked to surveys that gauge children's interest in and commitment to sports. It is noteworthy that Component 2 exhibits lower loadings compared to Component 1.Component 3 may correspond to several PAS components. Specifically, PAS1 (0.687), PAS2 (0.633), PAS3 (0.691), PAS4 (0.698), and PAS5 (0.705) show loadings on this component. It appears that Component 3 is associated with measuring the extent of encouragement young athletes receive from their parents. It is worth mentioning that Component 3 also includes certain loadings from items related to character development, but to a lesser extent. Measures of moral development, athletic engagement, and parental support can all be traced back to the study's three dimensions, which are Component 1, Component 2, and Component 3.Based on this analysis, Component 1, consisting of items focused on character development, appears to be the most significant. The second and third components are linked to sports participation and parental support, respectively, and they share some loadings with items assessing character development. This information illuminates the organization and interconnectedness of the survey questions, indicating that understanding the components of the research relies on three distinct factors: character development, sports involvement, and parental support. The results of the Kaiser-Meyer-Olkin (KMO) measure and Bartlett's Test of Sphericity are presented in Table 6, along with the Component Transformation Matrix.

**Table 6:** Component transformation matrix. KMO and Bartlett's estimates.

Component	1	2	3
1	0.997	0.061	0.043
2	−0.038	0.914	−0.405
3	0.064	−0.402	−0.913

**KMO and Bartlett's Test**		
Kaiser-Meyer-Olkin Measure of Sampling Adequacy.		0.976
Bartlett's Test of Sphericity	Approx. Chi-Square	13060.489
	df	435
	Sig.	0.000
**Model Fit Indices**		
Comparative Fit Index	CFI	0.95
Root Mean Square Error of Approximation	RMSEA	0.06

Squared multiple correlations, which indicate the proportion of variation in each item explained by the components, are presented on the diagonal (from the top left to the bottom right). Values outside the standard distribution reveal associations between variables and their constituents. The high squared multiple correlation of 0.997 in the first row and column suggests a strong relationship between the first item (likely associated with Component 1) and Component 1. Correlations between items and their constituents are represented by negative values in the off-diagonal elements. These numbers reflect the emphasis placed on specific components within the system.The data's readiness for factor analysis is assessed using the Kaiser-Meyer-Olkin (KMO) measure of sampling adequacy. A high KMO value, such as 0.976, indicates that factor analysis is likely to perform well with the data. This implies that the variables can be effectively summarized as factors because they share a significant amount of expected variation. To determine whether the correlation matrix significantly differs from an identity matrix (i.e., if the variables are interrelated), Bartlett's Test of Sphericity is employed. The test's very low p-value (Sig. 0.000) indicates that the correlation matrix is substantially different from the identity matrix, confirming the necessity for factor analysis by demonstrating the existence of relationships between the variables.

The fit indices, namely the CFI and the RMSEA, provide further evidence that the model is suitable. The CFI value of 0.95 and the RMSEA value of 0.06 demonstrate that the model fits the data well. Including these indicators makes the measurement model more reliable and valid. The AMOS software has performed the test. Table 7 focuses on the hierarchical regression analysis of character development mediation and moderation effects.

**Table 7:** Hierarchical regression estimates with mediation and moderation effects.

Model		Unstandardized Coefficients		Standardized Coefficients	t	Sig.	Collinearity Statistics	
		B	Std. Error	Beta			Tolerance	VIF
**Demographic Factors with Mediation Effects on Character Development**								
1	(Constant)	0.928	0.115	–	8.076	0.000	–	–
	Age	0.003	0.012	0.005	0.230	0.818	0.953	1.049
	Education	0.044	0.027	0.036	1.633	0.103	0.919	1.088
	Geography	−0.059	0.039	−0.032	−1.507	0.132	0.995	1.005
	Family structure	−0.059	0.029	−0.045	−2.047	0.041	0.933	1.072
	SEE	0.775	0.021	0.800	37.672	0.000	0.994	1.006
**Independent Variables with Mediation Effects on Character Development**								
2	(Constant)	0.284	0.096	–	2.960	0.003	–	–
	Age	0.005	0.009	0.008	0.475	0.635	0.946	1.057
	Education	−0.005	0.021	−0.004	−0.249	0.803	0.904	1.107
	Geography	−0.025	0.031	−0.013	−0.790	0.430	0.985	1.015
	Family structure	0.003	0.023	0.003	0.145	0.885	0.914	1.094
	SEE	0.174	0.032	0.180	5.425	0.000	0.256	3.900
	SPP	0.226	0.034	0.233	6.729	0.000	0.235	4.247
	PAS	0.135	0.034	0.140	3.978	0.000	0.227	4.398
	SPE	0.152	0.032	0.158	4.729	0.000	0.253	3.951
	PEI	0.244	0.030	0.254	8.074	0.000	0.284	3.526
**Mediation and Moderation Effects on Character Development**								
3	(Constant)	0.000	0.157	–	−0.001	0.999	–	–
	Age	0.006	0.009	0.011	0.635	0.525	0.942	1.062
	Education	0.012	0.023	0.010	0.525	0.600	0.804	1.244
	Geography	−0.024	0.031	−0.013	−0.777	0.438	0.985	1.015
	Family structure	0.009	0.023	0.007	0.393	0.695	0.903	1.107
	SEE	0.166	0.032	0.171	5.146	0.000	0.253	3.951
	SPP	0.218	0.034	0.224	6.447	0.000	0.232	4.301
	PAS	0.238	0.056	0.246	4.237	0.000	0.083	12.057
	SPE	0.143	0.032	0.148	4.420	0.000	0.249	4.012
	PEI	0.347	0.054	0.361	6.419	0.000	0.088	11.335
	SSN	−0.030	0.013	−0.184	−2.294	0.022	0.043	23.013

Model 1 examined how demographic factors affect character development after accounting for potential mediating influences. In Model 1, we aimed to study the impact of demographic variables on character development. One of the most prominent demographic trends is family composition, which can significantly influence an individual's life. Character development showed a significant negative correlation with family structure, particularly the presence of extended relatives. Participants from extended family structures statistically tended to exhibit less mature character, as evidenced by a t-value of −2.047, a probability value of 0.041, and a standardized beta coefficient of −0.045. Additionally, Model 1 included self-esteem as a moderating factor. The positive coefficient value for self-esteem (0.800, t-value = 37.672, prob. value = 0.000) underscores its centrality in moral growth. Individuals with higher self-esteem are more likely to exhibit positive character development. Self-esteem serves as a mediator between upbringing and personality development. While extended families may potentially have a negative impact on character development, the positive effects of a strong sense of identity can help mitigate this impact.

In Model 2 of the research, the focus transitioned from examining dependent variables to investigating how independent factors mediate character development through mediation. The findings revealed several important insights into the processes of character formation in contemporary Chinese adolescents. We found that the mediating effect of self-esteem was critical. The correlation between self-esteem and moral development was positive and statistically significant (coefficient 0.180, prob. value 0.000). This study suggests that individuals with higher self-esteem are more likely to exhibit enhanced character development. It underscores the significance of the link between youth sports involvement and positive self-perception and self-worth [71]. There was a significant positive correlation between sports participation and character maturation (coefficient 0.233, prob. value 0.000). A significant positive correlation was also found between character development and the availability and quality of sports facilities. Juveniles' access to safe, well-maintained sports facilities is statistically correlated with positive character development (coefficient 0.158, prob. value 0.000). This finding underscores the significance of the context in shaping character. Finally, the coefficient for the peer influence variable was 0.10254 (prob. value 0.000), indicating a positive relationship between peer influence and personal growth.

In our third and final model, Model III, we looked into the intricate network of mediation and moderation effects to better understand how different factors interact and contribute to character development in Chinese youth. We observed that self-esteem mediated the relationship between moral growth and academic success. A positive and statistically significant correlation was found (coefficient = 0.171, probability = 0.000). These results emphasize the importance of self-esteem in moulding one's personality. There is a negative interaction between social support nexus and character development, as shown by the coefficient value of −0.184 (t-value: 2.294, prob. value: 0.022). There are significant ramifications of this result.

Goodness-of-fit estimates for the various models considered are summarized in Table 8. These estimates shed light on how well the models capture the complexities of the interplay between factors.

**Table 8:** Goodness-of-fit estimates.

Model	R Square	Adjusted R Square	Std. Error of the Estimate	Change Statistics					Durbin-Watson
				R Square Change	F Change	df1	df2	Sig. F Change	
1	0.640	0.638	0.52901	0.640	285.329	5	802	0.000	
2	0.776	0.773	0.41870	0.136	120.558	4	798	0.000	
3	0.777	0.774	0.41759	0.001	5.262	1	797	0.022	1.663

The R-squared value of 0.640 for Model 1 indicates that the independent factors account for 64 % of the variation in CHD. The Adjusted R-squared is 0.638, taking into account the number of predictors and the sample size. The precision of these predictions is reflected in a Standard Error Estimate of 0.529. A Durbin-Watson test of autocorrelation in the residuals yielded a score of 1.663, indicating that autocorrelation is not present at a statistically significant level.In Model 2, the R-squared value increases to 0.776, suggesting that when the mediating variable (Self-Esteem) is included, 77.6 % of the variation in character growth is explained. The Adjusted R-squared is 0.773, still considering the number of predictors and sample size. If the estimate's standard error drops below 0.41870, it means that more precise predictions can be made. The F Change statistic is 120.558 with 4 and 798 degrees of freedom, indicating a significant improvement in model fit, while the R-squared metric has increased by 0.136.Model 3 introduces the moderating variable (Parental Support x Peer Influence), which increases the R-squared to 0.777 and the Adjusted R-squared to 0.774. The estimate's standard error is now 0.41759. While the Change Statistics show only a 0.001-point improvement in R-squared, the F Change statistic of 5.262 (with 1 and 797 degrees of freedom) suggests a significant improvement in model fit. These results provide further evidence that the model accurately represents the interdependencies between the variables of interest. The Analysis of Variance (ANOVA) estimates for the various models in the study are presented in Table 9.

**Table 9:** ANOVA estimates.

Model		Sum of Squares	df	Mean Square	F	Sig.
1	Regression	399.252	5	79.850^a^	285.329	0.000^b^
	Residual	224.443	802	0.280		
	Total	623.694	807	–		
2	Regression	483.794	9	53.755	306.621	0.000^c^
	Residual	139.900	798	0.175		
	Total	623.694	807	–		
3	Regression	484.711	10	48.471	277.959	0.000^d^
	Residual	138.983	797	0.174		
	Total	623.694	807	–		

a Dependent Variable: CHD.

b Shows first model.

c Shows second model, and.

d Shows third model of the study.

Model 1, which includes both demographic characteristics and mediation effects, has a sum of squares (SS) of 399.252 for its regression analysis. It has a mean square (MS) of 79.850 and 5 degrees of freedom (df). This model significantly explains the variation in CHD, as demonstrated by the F-statistic of 285.329 and the accompanying p-value of less than 0.001 (p < 0.001).The regression SS for Model 2, which includes independent variables with mediation effects, is 483.794 (9 df). The F-statistic is 306.621, and the MS is 53.755. This model also substantially explains the variation in CHD, as evidenced by the p-value being less than 0.001 (p < 0.001).The regression SS for Model 3, with the moderation variable added, is 484.711 for a total of 10 degrees of freedom. The F-statistic is 277.959, and the mean squared error is 48.471. Because the p-value is less than 0.001 (p < 0.001), we can conclude that this model significantly improves upon previous attempts and adequately explains the observed variation in CHD.

All three models have highly significant p-values, suggesting that they effectively account for the observed data about character development.

## 5 Discussions

Model-I raises questions about why extended family arrangements, which are common in the demographic survey, are associated with lower character development. The increased number of relatives in extended families leads to more complex social dynamics, potentially resulting in various social constraints and role expectations that may impact character development [72]. While family members of different ages can be valuable resources, this research suggests that there may be drawbacks to growing up in a large family. It is important to note that the correlation between family dynamics and personality maturation does not imply causation [73]. Individuals from strong extended family structures may better handle the challenges inherent in their family relationships and grow as individuals as a result [74]. Another interesting finding from this survey is the prevalence of multigenerational households, ranking third after nuclear families and single-parent homes. These findings highlight the need for research on the interplay between self-esteem and family dynamics in relation to character development. Insights like these can help shape programs and services aimed at helping young people, especially those in larger families, develop positive character traits.

Model-II indicates that as children's participation in sports activities increases in frequency and intensity, character development is likely to improve. It supports the notion that sports participation plays a crucial role in developing desirable personality traits and practical skills [75]. Another significant predictor of character maturation was parental encouragement (coefficient 0.140, prob. value 0.000). It highlights the importance of parental support for their children's athletic endeavours. Strong parental encouragement positively affects children's character development, emphasizing the role of the family environment [76]. This suggests that when young individuals are encouraged by their peers to engage in sports, it can have a positive impact on their character development. As noted by Espenberger et al. [77], the effect of one's social network on sports involvement should be taken into account in character development methods.

Model –III shows that self-esteem may be favourably impacted by their participation in sports, parental support, access to sports facilities, and peer influence [78]. This can have a ripple effect on the child's character development. The findings of this research support the idea that treatments that help young people feel good about themselves might have a favorable impact on their character development, especially among Chinese youths who participate in sports. Parental support and peer pressure were shown to have a significant effect on developing a person's character. While both parental participation and peer influence may have beneficial impacts on a youth's character development individually, the findings imply that their combined impact tends to lessen these benefits [79]. When children have strong relationships with both parents, positive peer pressure has less influence on their personal growth. This may imply that children who grow up with ample positive reinforcement from their parents are better able to withstand the influence of their peers. This study underscores the importance of parents in shaping their children's identities despite exposure to negative peer pressure. Encouraging parents to take an active role in their children's athletic activities and emphasizing the value of positive character traits can offset the adverse effects of peer influence [80].

The study's overarching goal was to show how self-esteem mediates the relationship between athletic involvement and beneficial developmental outcomes. Theoretically, we drew on Social Cognitive Theory and Self-Determination Theory to postulate that participating in sports increases one's self-esteem by encouraging a feeling of mastery, independence, and belonging. The study aimed to determine, using multivariate regression and mediation analysis, how much self-esteem mediates the association between participation in sports and various outcomes, such as character development, cooperation, and life skills. The research aims to shed light on the psychological processes that underlie the positive impacts of juvenile sports on personal development and well-being by analyzing these pathways. In addition, the study looked at how parental encouragement and support acted as a moderator in these models. Given parents' significant influence on their children's growth and development, the study postulated that characteristics related to parents may interact with their children's involvement in sports to either increase or decrease participation affects outcomes. The hypothesis was that self-esteem would benefit even more from sports participation if their parents provided positive reinforcement through praise, positive reinforcement, and good role modeling. On the other hand, if parents do not support or encourage their children, it could be more challenging for sports to benefit their development. The goal of conducting moderation studies and interaction effects was to discover how parental variables influence the association between young sports participation and overall development in a complex fashion.

## 6 Conclusions and policy implications

In the context of Chinese youth, this research unravels the intricate web of connections between youth sports participation, moral development, and skill acquisition. Analyzing data from a demographically representative sample of 808 individuals, we were able to illuminate the various ways in which family background, personal attributes, and other independent variables all contribute to shaping an individual's personality and behaviour. This research offers insights into the complex dynamics involved in the development of today's Chinese youth, highlighting the crucial role of parental guidance in character formation in the face of peer pressure.

The following policy recommendations emerge for stakeholders involved in Chinese youth development programs. Firstly, we emphasize the importance of promoting Family Support. Encouraging parents to support their children's athletic pursuits actively and instil positive character traits is essential. Fostering holistic youth development can be achieved by encouraging parents to engage in meaningful conversations with their children about the value of character strengths. Simultaneously, it is crucial to prioritize the development of youth sports infrastructure. It is recommended that resources be allocated to construct and maintain sports facilities that are accessible and affordable in both urban and rural areas. Consequently, opportunities for youth sports participation will be more widely available and accessible.Moreover, the Education System should make character education a central pillar. Schools should consider incorporating character education courses into their regular curricula. By placing equal emphasis on the teaching of strong character attributes alongside traditional academic disciplines, we can instill morals and ethics in the next generation. The impact of Peer Education on character development should be considered. In light of our findings, peer-led educational programs are highly recommended. These programs can be developed due to their ability to harness the motivating influence of peers. Youth character development can be positively influenced when peers serve as mentors and leaders within sports teams.

Interventions aimed at boosting self-esteem are crucial, given the central role self-esteem plays in this process. Sports should be reframed as opportunities to discover oneself and enhance one's abilities. Additionally, organizing workshops for parents may yield positive results. Equipping parents with the knowledge and skills to guide their children through athletic experiences and fostering a strong awareness of the importance of character development can be transformative.Lastly, it is essential to conduct ongoing program evaluations. Youth sports programs should be continually monitored and assessed for their impact on character development. Regular assessments will enable timely adjustments and enhancements, keeping these initiatives responsive to the evolving needs of Chinese adolescents.The lives of young people in China could significantly improve if the government were to implement these comprehensive recommendations. A brighter and more secure future awaits the next generation if they can enhance their capacity for character development, life skill acquisition, and resilience in the face of adversity.

Recognizing such constraints in our research is crucial. One limitation is that we may have recruited individuals from an undersized subset of the young Chinese population, which increases the likelihood of sampling biases and reduces the generalizability of our results. Furthermore, the data acquired may need to be more accurate or reliable because of response biases or social desirability effects introduced by relying on a small number of scale measurements. The research may also be limited by the methodological restrictions of cross-sectional designs, which prevent us from establishing causation or temporal correlations between variables, even if we used statistically solid approaches to examine the data. Using more varied and representative populations, objective metrics, and longitudinal or experimental methods to improve the validity and generalizability of results should help future research overcome these constraints. Our goal in mentioning these possible limitations is to be forthright and fair in our analysis of the study results and to point the way toward areas that may be improved upon in the future.

## Funding statement

This research was supported by Hunan Province Project Identification Number: XJK22CTW028 (Practical study of physical education course reform in junior middle school in the context of “Healthy China").

## Ethics declaration

This study protocol was reviewed and approved by the Ethical Committee of Hunan University of Technology with approval number: HNUT-IRB-2022-070325. All the information of the participants was hidden study and the informed consent was obtained from all the participants with the approval of the Ethical Committee of Hunan University of Technology.

## Data availability statement

Data will be made available on request.

## CRediT authorship contribution statement

**Kai Yi:** Data curation, Conceptualization. **Han Luo:** Methodology, Formal analysis. **Lihong Wei:** Writing – original draft, Supervision.

## Declaration of competing interest

The authors declare that they have no known competing financial interests or personal relationships that could have appeared to influence the work reported in this paper.
